# Single pullout experiment and reinforcement properties of basalt fiber in vegetation concrete

**DOI:** 10.1038/s41598-021-04760-0

**Published:** 2022-01-24

**Authors:** Baohua Zhang, Daxiang Liu, Huaizhi Su

**Affiliations:** 1 State Key Laboratory of Hydrology-Water Resources and Hydraulic Engineering, Hohai University, Nanjing, 210098 China; 2 College of Water Conservancy and Hydropower Engineering, Hohai University, Nanjing, 210098 China; 3 Key Laboratory of Disaster Prevention and Mitigation, (China Three Gorges University), Hubei Province, Yichang, 443002 China; 4 Key Laboratory of Geological Hazards On Three Gorges Reservoir Area (China Three Gorges University), Ministry of Education, Yichang, 443002 China

**Keywords:** Civil engineering, Structural materials

## Abstract

Basalt fiber (BF) reinforced vegetation concrete (VC) technique has attracted the attention of researchers. In order to investigate the reinforcement properties of BF reinforced VC, the optimal BF length and content. Through the single BF pullout test and direct shear test, the properties of interfacial strength between BF/VC and the reliability of the formula for calculating the optimal BF reinforcement length are studied. It has been found that the designed equipment is an efficient method to obtain the interfacial peak shear strength and residual shear strength of BF/VC. Moreover, the direct shear test proves the feasibility of the formula, which is used as a basis for mixing BF length in engineering. The anchoring effect between the cement hydration product and the fiber in the VC changes the mechanical action between BF/VC and significantly improves the shear strength of the interface. Higher dry density effectively enhanced the peak tension of a single BF by 149.23%. The optimal BF length and content make the softening degree of vegetation concrete not evident, which improves the durability of VC engineering. The formula of optimum fiber reinforced length and empirical formula can be used as reference for mixing basalt fiber in practical engineering.

## Introduction

Ecological restoration is an important issue in global environmental protection^1–3^. As engineering construction moves into new areas, ecological restoration becomes an issue that must be solved^4–7^. The harsh engineering environment has made ecological restoration more difficult, with the most difficult ecological restoration of the slopes. Vegetation concrete (VC) grows plants on rocky slopes and improves slope stability^8,9^. In recent years, VC has been widely used in China. It is a useful method of improving slope ecology. However, VC strength decreases, and cracks expand rapidly in cold areas. Therefore, more long-term ecological restoration results have been obtained by improving the strength and durability of VC.

It is generally known that fiber is an engineering reinforcement material, making an important role in improving the performance of construction materials^10–14^. That randomly distributed fibers reinforce soil, composite soils and concrete have recently attracted increasing attention in civil engineering not only in the scientific research environment, but also in projects, including dams, railways, and slopes^15–19^. A large number of experiments have been conducted to investigate the mechanical behavior of fiber-reinforced concrete and soil, including the triaxial compression test, unconfined compression test, and freeze–thaw test^20–25^. These studies have shown that fiber improves the durability and strength of concrete and soil. The main reasons for the strength and durability of the material are the tensile strength of a single fiber and the fabric group structure. As pointed out by Tang et al.^13,26^, who carried out a single polypropylene fiber pullout test and observed that different soil dry density and water content can result in different pullout resistance of fiber from the soil. And investigated the pullout resistance significantly depends on the interfacial contact condition with soil. Many scholars found that the interfacial shear strength was a critical factor that controls the toughness and mechanical properties of composite materials^27–31^. Therefore, fiber type and fiber strength were important factors that affected the interfacial shear strength in the same composite matrix.

Basalt fiber (BF), as a new environmental-friendly high-strength fiber material, can improve the strength and durability of soil^32–37^. Moreover, the related researches show that BF enhances the toughness, impermeability, and preventing cracks of concrete^38–41^. Zhou^42^ indicated that BF enhanced effect on the compressive strength, tensile strength, and flexural strength. The improvement effect was the highest with the basalt fiber content was 0.3% and 0.4%. The test results demonstrate that the reinforcement effect is different with differences length and content of BF. However, BF has not been applied in VC. The reinforcing properties of BF in VC are still not fully understood.

In this paper, BF was added to the VC to improve strength and durability. The optimal BF reinforcement length formula was obtained by mechanical hypothesis. A single basalt fiber pullout test was using self-made equipment. The effects of water content, dry density, and cement hydration products on the interfacial mechanical properties of BF/VC and pullout response were investigated. The optimal BF reinforcement length and content were obtained by the direct shear test. Moreover, the properties of BF reinforcement and the reliability of the theory of optimal BF reinforcement length were discussed.

## Materials and method

### Materials

**Figure 1:**
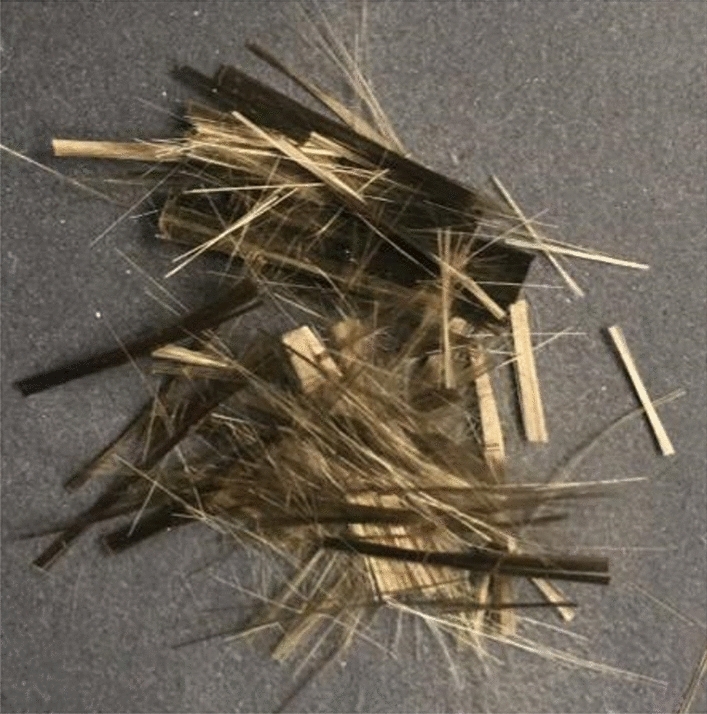
Basalt fibers.

Vegetation concrete (VC) was prepared by mixing clay, cement, organic material, microbial agent, and water at a weight ratio of 100:8:6:4. The clay was collected from a foundation in Yichang, Hubei. It was retrieved, dried, mashed, and sieved through a 2 mm sieve. The P.C 32.5 cement used was produced by Huaxin Cement Co., LTD. organic material, which was prepared from local fir sawdust, was dried in an oven first, and then sieved through a 2 mm sieve. The physical properties of the basalt fiber (BF) provided by the manufacturer are given in Table 1. The microbial agent used was a patented product provided by China Three Gorges University. The BF was shown in Fig. 1.

**Table 1:** Physical properties of the BF.

Parameter	Value
Fiber type	Basalt fiber
Section length (μm)	≥ 600
Section width (μm)	≥ 86
Breaking tensile strength(MPa)	≥ 105
Elongation at break (%)	≤ 3.1
Modulus of elasticity(GPa)	40
Acid- and alkaline-resistance	pole-strength

## Method

### Preparation of the test samples

The composition ratios of VC used in the experiment were as follows: the weights of the cement, organic material, and microbial agent were 8%, 6%, and 4% of the clay. The optimum moisture content, plastic limit, liquid limit, and maximum dry density of VC were 20%, 23.6%, 44.57%, and 1.57 g/cm^3^ by the test.

**Figure 2:**
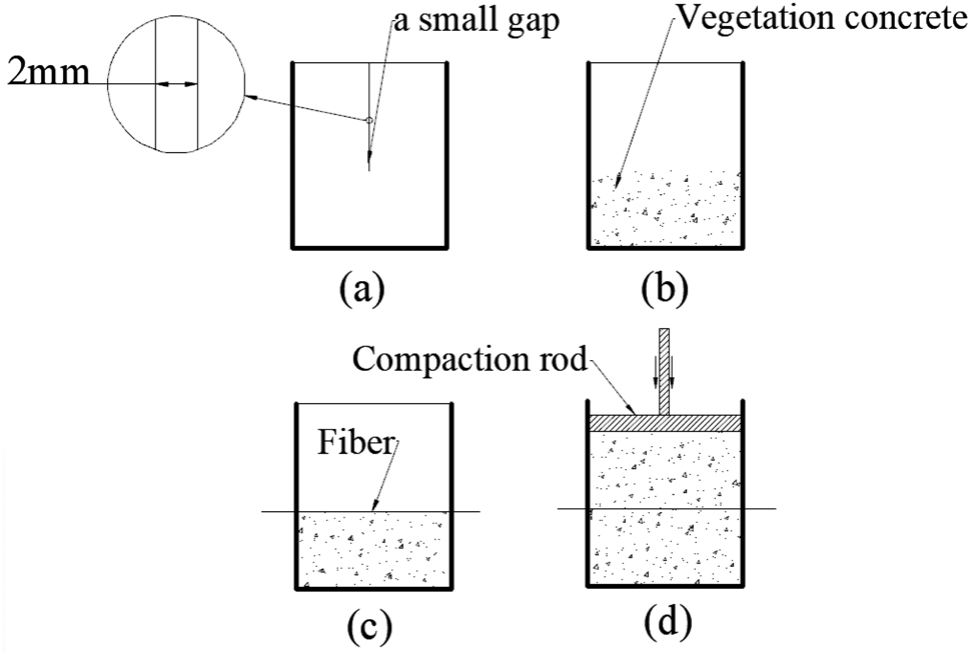
Sample preparing process (not to scale).

Test samples (5 × 5 × 5 mm in dimension) used in the single BF pullout test were compacted in mold shown in Fig. 2. The inside diameter of the mold was 5 × 5 × 5 mm, two small gaps (2.5 mm in length and 1 mm in width) were formed on the opposite sidewalls of the mold to facilitate fiber placement during compaction. To assess the effect of dry density and water content on the pullout behavior of BF, test samples’ names and parameters were shown in Table 2. The samples preparation process described as follows in Fig. 2 :(a) half of the quantity of VC was put in the mold, and flatted the surface; (b) one BF was placed through the gaps of the mold on the surface, and the other half of the VC was put into the mold; (c) VC was compacted by the compaction rod to test density;(d) the sample was demoulded and wrapped in a preservative film. In the single fiber pullout test, 9 groups of samples were prepared, with 3 parallel samples for each group. All samples were cured for 7 days under standard curing conditions until tests.

**Table 2:** Parameters of pullout test samples.

Sample No	Water content *ω*(%)	Dry density *ρ*_d_(g/cm^3^)
S11	18	1.35
S12	18	1.45
S13	18	1.55
S21	20	1.35
S22	20	1.45
S23	20	1.55
S31	22	1.35
S32	22	1.45
S33	22	1.55

The direct shear samples (Φ61.8 × 20 mm) were made by cutting ring. First, the vegetated concrete and basalt fibers were mixed. Then samples were directly prepared by the cutting ring. The water content of the samples was 20% and the dry density was 1.45 g/cm^3^. Details of samples were indicated in Table 3. All of the samples were wrapped by plastic wrap and cured 7 days under standard curing conditions until tests.

**Table 3:** Parameters of direct shear samples.

Sample no	Fiber length (mm)	Fiber content (wt.%)	Fiber class
D00	0	0	-
D11	6	0.05	Basalt fiber
D12	6	0.35	Basalt fiber
D13	6	0.65	Basalt fiber
D21	9	0.05	Basalt fiber
D22	9	0.35	Basalt fiber
D23	9	0.65	Basalt fiber
D31	12	0.05	Basalt fiber
D32	12	0.35	Basalt fiber
D33	12	0.65	Basalt fiber

#### Apparatus and test

**Figure 3:**
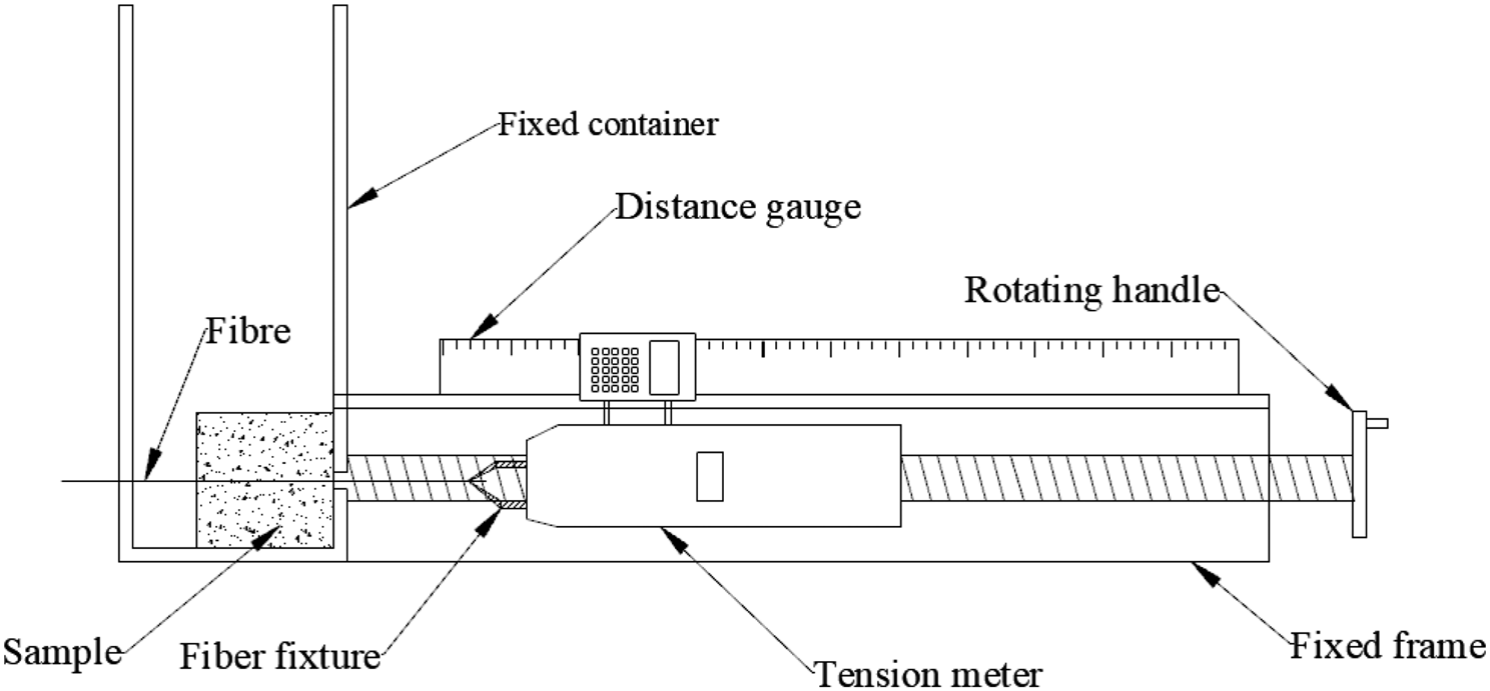
Single basalt fiber pullout apparatus (not to scale).

The apparatus for the single basalt fiber pullout test is illustrated in Fig. 3. It is basically composed of HP-50 Adberg digital display gauge (measuring range: 50 N, resolution: 0.01 N), displacement sensor, fixed container, fixture, pulling device, and other required instrumentations. As shown in Fig. 3, the single fiber sample was laid on the left of the fixed container with one free end of fiber through the opening of the container, and secured with the fixture. During the test, the handle was rotated uniformly to generate tensile load on the fiber until the fiber is broken or pulled out of the VC. The video was used to record, that ensure the clear index of the tension meter and electronic displacement meter during the process. The handle rotation was controlled at 1.5 mm/min. The tension and displacement are recorded in excel by video.

In order to quantify the interfacial mechanical behavior between the BF and the VC, interfacial peak shear strength ($\tau_{f}$) and interfacial residual shear strength ($\tau_{r}$) are introduced and defined as follows:

1 $$\tau_{f} = \frac{{N_{max} }}{S}$$

2 $$\tau_{r} = \frac{{N_{r} }}{S}$$

where $N_{max}$ is the maximum load before the interface shear failure, $N_{r}$ is the residual load applied on the BF, $S$ is interfacial contact surface between the BF and the VC, which can be calculated based on the actual embedded BF length and sectional area. In this study, the BF embedded length is 5 mm.

**Figure 4:**
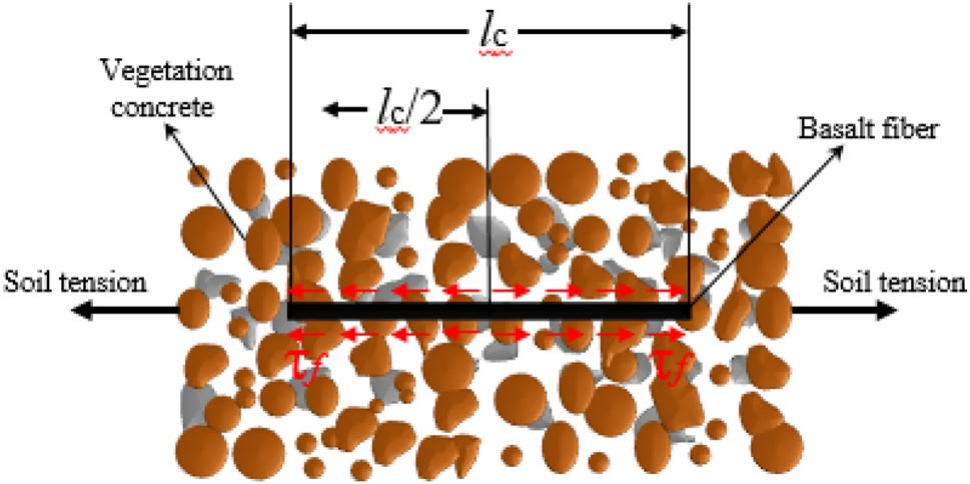
Sketch drawing of the principle for the optimal BF reinforcement length.

The optimal BF reinforcement length is beneficial to the effective use of BF strength and the reduction of material waste in practical engineering. According to the research results^13,43^, this paper preliminarily establishes the assumption that the fibers are laid in a straight line in the soil, the tension cracks occur at the middle point of the fibers and a single BF is a rectangular prism of flat as shown in Fig. 4. The optimal BF reinforcement length ($l_{c}$) is calculated as follows:

3 $$l_{c} = \frac{{R_{f} ab}}{{\left( {a + b} \right)\tau_{f} }}$$

While $R_{f}$ is the BF tensile strength (105 MPa), a is the BF section length (0.61 mm), b is the BF section width (0.086 mm).

**Figure 5:**
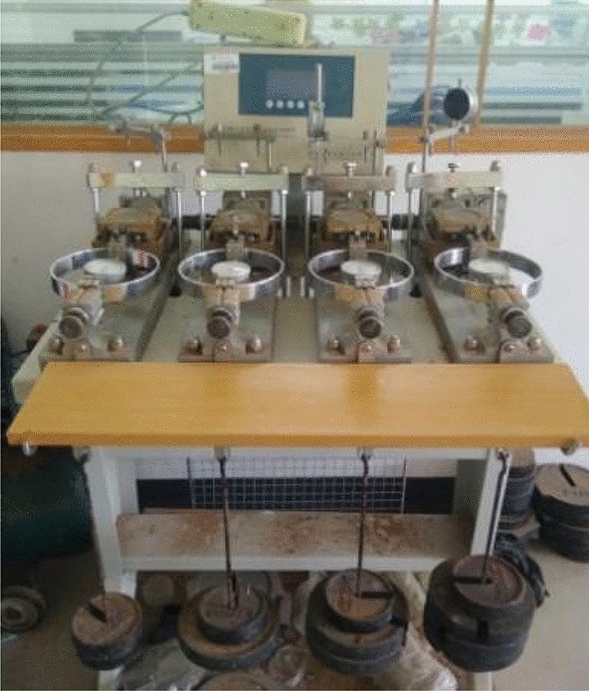
ZJ strain-controlled direct shear apparatus.

The shear strength of samples is measured by the strain-controlled direct shear apparatus (which was produced Nanjing Soil Instrument Factory) (Fig. 5), with the computer controls the strain rate (0.8 mm/min) and simultaneously loads at four normal stresses (100 kPa, 200 kPa, 300 kPa, and 400 kPa). The direct shear test obtained the shear strength (τ), cohesion (c), and internal friction angle (φ) of the samples, and conditioned the optimal BF reinforcement length and a mass ratio of the BF.

## Results and analysis

### Pullout test characteristics of the single basalt fiber

#### Characteristics of the pullout

**Figure 6:**
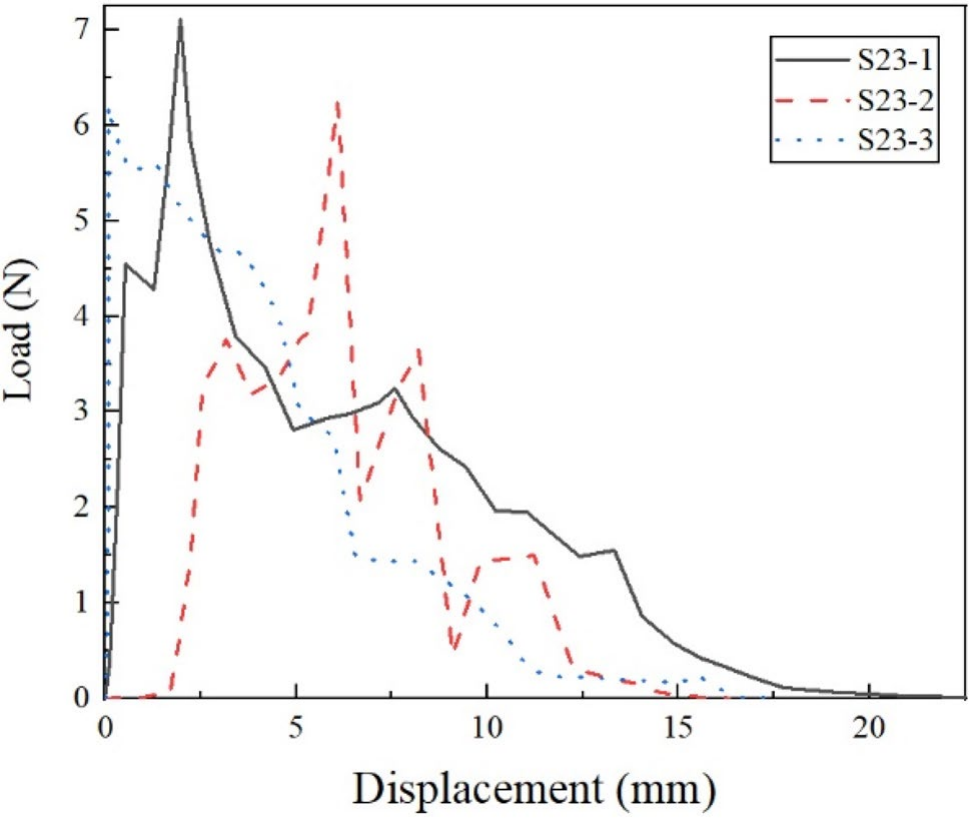
pullout load–displacement curves of the three parallel samples compacted at 20% water content and 1.55 g/cm^3^ dry density.

**Figure 7:**
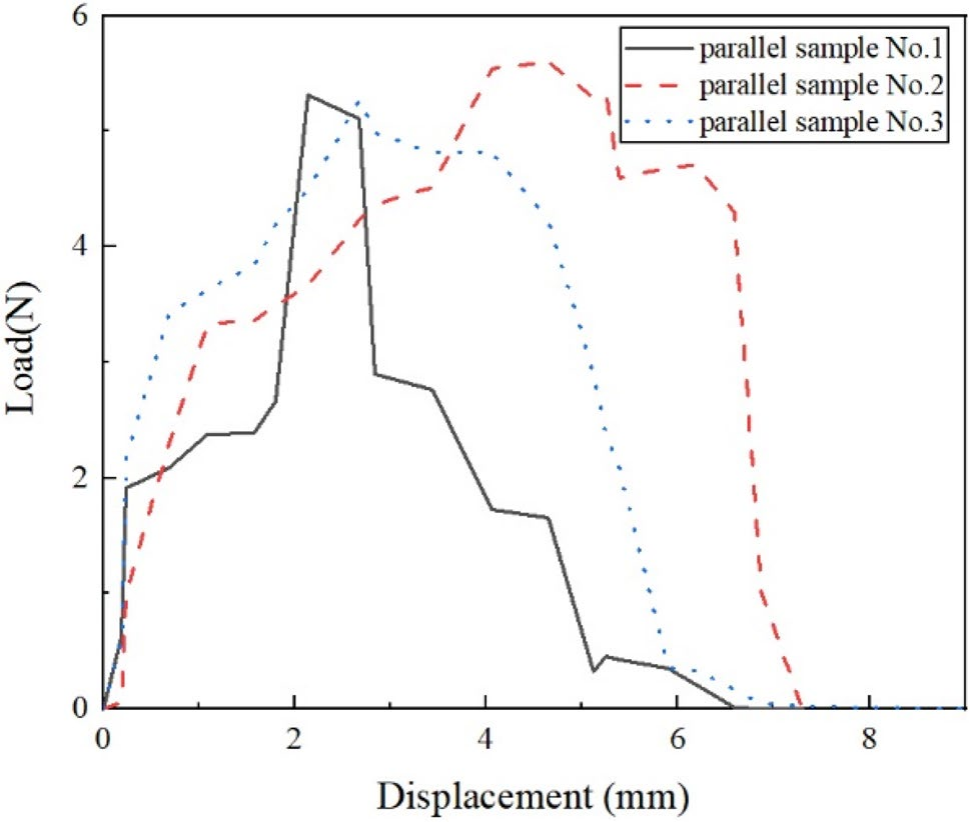
pullout load–displacement curves of the fiber in clay.

**Figure 8:**
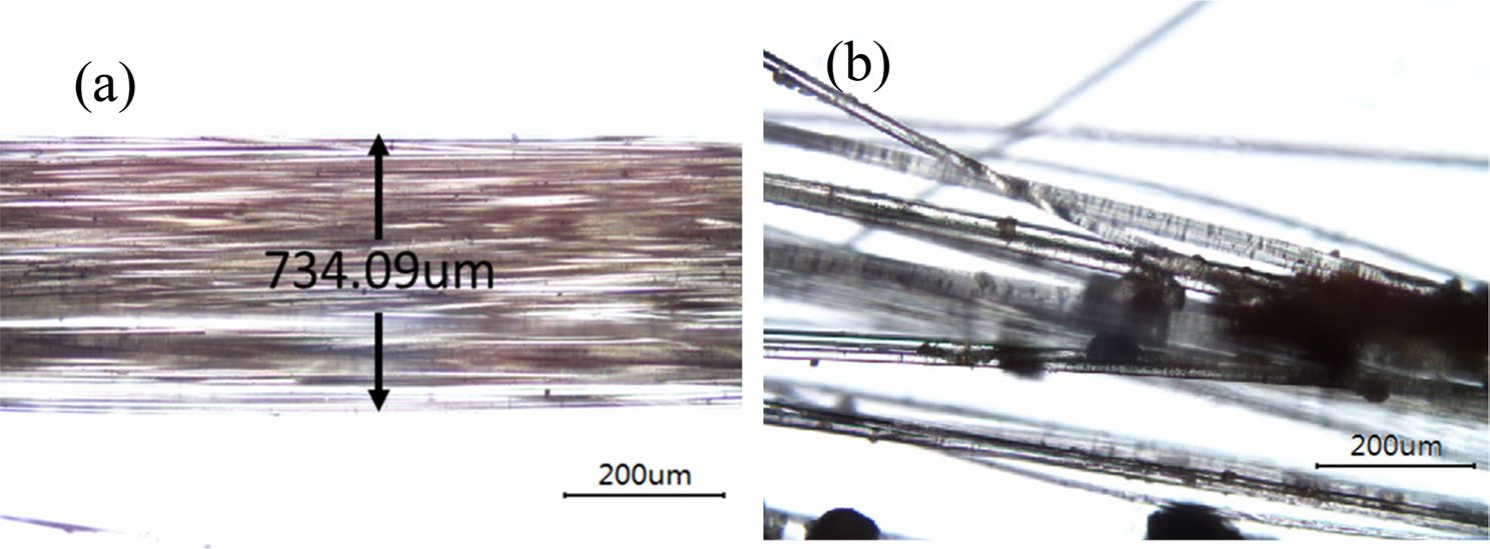
The sample polarizing microscope photos. (**a**) Basalt fiber before the test. (**b**) Basalt fiber surface attachment after the test.

**Figure 9:**
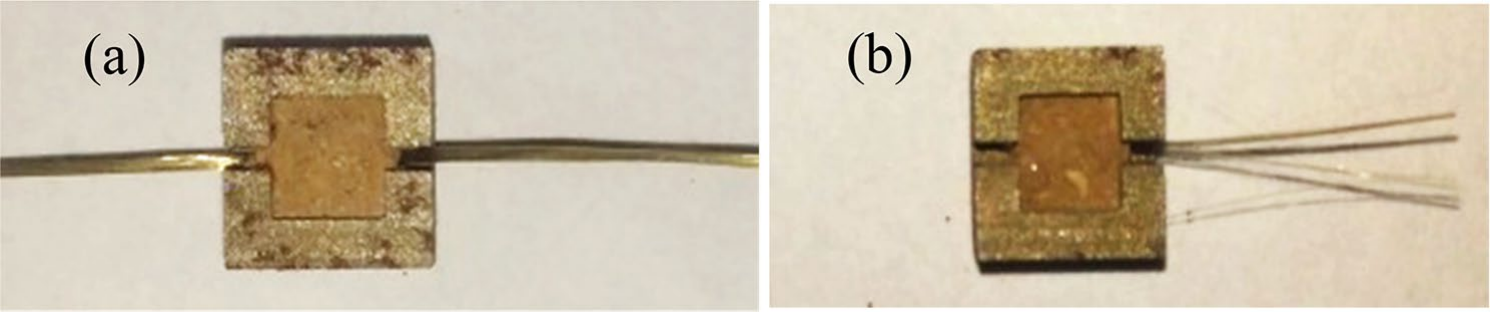
The sample photos. (**a**) Basalt fiber before the test. (**b**) Basalt fiber surface attachment after the test.

Figure 6 shows the pullout load–displacement curves of the three parallel samples at the optimum moisture content (20%) and dry density (1.55 g/cm^3^) in VC. It can be seen that the results obtained on the parallel samples are similar, and the repeatability is good. The results show that the applied load increases quickly with increasing pullout displacement, which drops in a typical multi-peaks pattern after a peak load is reached. Figure 7 shows the pullout load–displacement curves of the BF in clay, which is significantly different from that of VC. The curves did not have multi-peaks in clay after a peak load. Tang and Zhang^43,44^ found that the process of single fiber pullout leads to shear stress at the interface. During the test, the pullout load stores in the lower free end of the fiber and interface in the form of strain energy. When the pullout load reaches the peak value, which corresponds to the interfacial shear strength, interface shear failure occurs. The strain energy is released, resulting in the subsequent decrease of pullout load. As the fiber is straight and smooth, the sliding friction between fiber and clay is relatively low and generally constant. However, the curves show multi-peak characteristics in the vegetation concrete (VC), which is related to the composition of the VC. The composition of the VC changes the mechanical properties of the interface between fiber and matrix, and is reflected in the pullout curve. The cement hydration products wrap around the fibers in VC. When the BF is in the test, in addition to the frictional and cohesive forces, there is the anchoring of the BF by the cement hydration products. The hydration products are unevenly distributed in the vegetated concrete, so there are multiple anchoring zones on the fiber. The fiber is pulled off when the load is sufficient to overcome the anchoring force. Then the tension is transmitted to the next anchoring section and load rises. This results in multi-peaks characteristics. It is interesting to find that peak value gradually decreases with decreasing load. To explore the reason lying behind this, we used a camera and a polarizing microscope to record the characteristics of the BF before and after the test (Fig. 8 and 9). According to pictures and experimental phenomena show the following conclusions: (a) The BF change from clusters to dispersions. This is because the BF is broken down one by one during the test, which results in a decrease in the peak value of the drop. (b) There are many attachments on the surface after the test, which indicate that there is not only friction but also cementation between the BF and VC.

### Effect of water content

**Figure 10:**
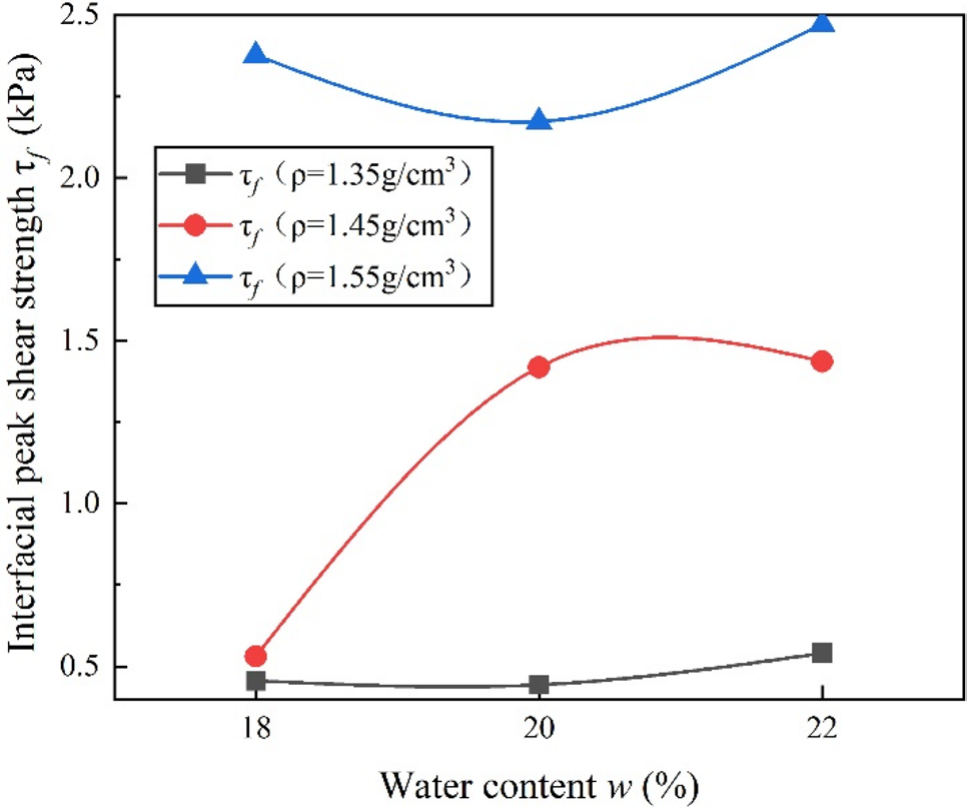
Variation of interfacial peak shear strength for BF reinforced samples compacted at different water contents and density.

Water content makes a key role in BF/VC interfacial shear strength as shown in Fig. 10. The results show that the dry density 1.55 g/cm^3^ samples have the lowest $\tau_{f}$ at the optimal moisture content(i.e., *w* = 20%). The $\tau_{f}$ of dry density 1.35 g/cm^3^ and 1.45 g/cm^3^ samples increase with increasing water content. The main reason for this difference is that the BF/VC interfacial shear strength is composed of the anchoring force of cement hydrate, interfacial friction and interfacial cohesion, which are related to water content. The $\tau_{f}$ of the samples with maximum water content is higher than the minimum water content. Increased water content contributes to improved hydration of cement in VC resulting in increased peak shear strength. The reason for the difference is that the samples with the highest dry density (*ρ*_d_ = 1.55 g/cm^3^) have the lowest porosity, the higher water content reduces the matrix suction, and effective stress between particles. The bonding strength and friction between adjacent particles are therefore decreased. As a result, particles on the shear interface may be easy to be disturbed and rearranged during the pullout process, and the interfacial resistance to shear is weakened. The water content of the sample is higher than the optimum water content, and excessive water reacts with more cement. Hydration enhances the cohesion and anchorage between the particles, thus increasing the BF/VC interfacial shear strength.

### Effect of dry density

From Fig. 10, it is found that $\tau_{f}$ increases by 149.23% on average with the increase of dry density. The higher dry density with the same water content, the greater the anchoring force is caused by more cement hydration products. The higher dry density of the samples corresponds to a lower void ratio and smaller pore diameter. It means that the effective interfacial contact area between fiber and matrix increases with increasing dry density, which improves the interfacial bond strength and the associated friction. The higher dry density of the samples, the higher the covering mass of the same thickness on the BF, the higher the vertical stress of the BF given by the covering, thus improving interfacial friction. In the process of specimen preparation, more compaction work or normal stress should be applied to obtain higher dry density and results in higher contact force and interlock between adjacent particles. As mentioned previously, this behavior will also enhance the interfacial shear resistance.

## Empirical formulation

### Establishing an empirical formulation

The interfacial shear strength of BF/VC is mainly affected by water content and dry density. They are also important factors affecting the restoration when VC is used in the actual ecological restoration project. The empirical formula of optimal BF reinforcement length is assumed as follows:

4 $${\text{l}}_{{{\text{max}}}} {\text{ = kw}}^{{\text{e}}} {\uprho }^{{\text{f}}}$$

While $l_{max}$ (mm) is the optimal BF reinforcement length, $w$ is water content, $\rho$ is dry density, k, e and f are constant terms.

The logarithm of both sides are written as follows:

5 $$\lg l_{{\max }} = \lg {\text{k}} + e\lg w + {\text{f}}\lg \rho$$

Set *Y* = ${\text{ lg}}l_{max}$, *m* = ${\text{ lgk}}$, *X*_1_ = ${\text{lg}}\omega$, and *X*_2_ = ${\text{lg}}\rho$ are substituted into the Eq. (5), which is written as follows:

6 $${\text{Y = m + eX}}_{{1}} {\text{ + fX}}_{{2}}$$

The unknown coefficients m, e and f of multiple linear regression equation are solved by substituting test data into the Eq. (6).

### Solving empirical formulas

Multiple linear regression equation is analyzed and solved by spss22.0. The *R*^2^ = 0.900 and significance < 0.05, indicating that the definition of the formula is reasonable. The Spss 22.0 calculate m = 5.278, e = -2.011, and f = -11.478. The Eq. (6) is written as follows:

7 $${\text{Y = 5}}{.278 - 2}{\text{.011X}}_{{1}} { - 11}{\text{.478X}}_{{2}}$$

Set *Y* = ${\text{ lg}}l_{max}$, *m* = ${\text{ lgk}}$, *X*_1_ = ${\text{lg}}\omega$, and *X*_2_ = ${\text{lg}}\rho$ are substituted into the Eq. (7), which is written as follows:

8 $$\lg l_{{\max }} = 5.278 - 2.011\lg w - 11.47\lg \rho$$

Remove logarithms from both sides of the Eq. (8) is written as follows:

9 $${\text{l}}_{{{\text{max}}}} { = 10}^{{{5}{\text{.278}}}} {\text{w}}^{{{ - 2}{\text{.011}}}} {\uprho }^{{{ - 11}{\text{.47}}}}$$

Equation (9) is the empirical formula. The optimal BF reinforcement length (6.46 mm) is obtained by substituting the water content (*w* = 20%) and dry density (1.45 g/cm^3^) of the direct shear test design into the Eq. (9).

### Shearing strength behavior of BF reinforced VC

#### Shearing strength-displacement curves

**Figure 11:**
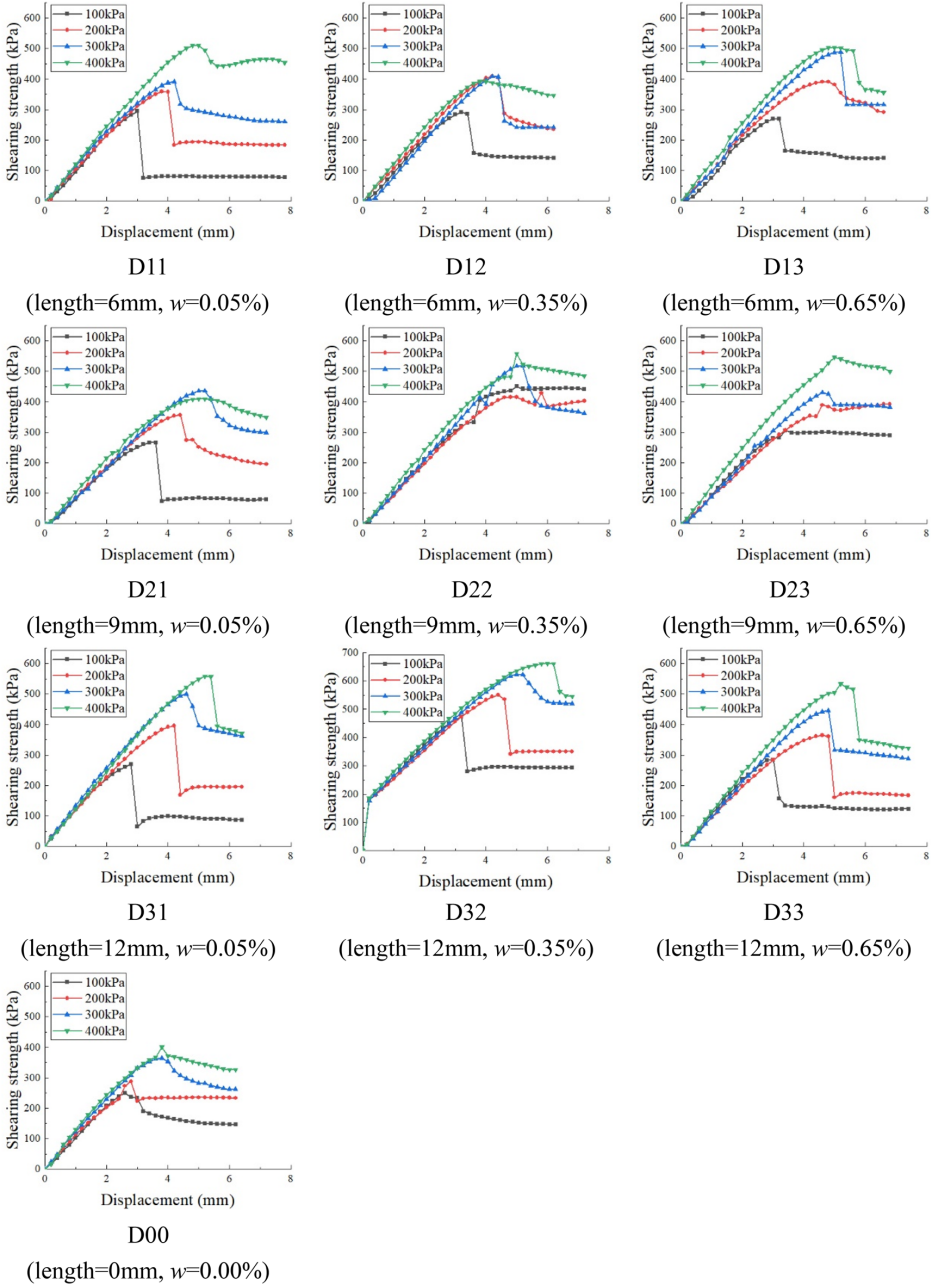
The shearing strength-displacement curves under different normal stress.

The shear strength of samples is measured by the direct shear apparatus. The shearing rate was 0.8 mm/min under four normal stresses. Figure 11 shows the shearing strength-displacement curves of samples with normal stresses of N = 100 kPa, 200 kPa, 300 kPa, and 400 kPa. Most of the curves show softening behavior except D22 and D23.In the previous article, it is found that the BF mainly affects the cohesion, friction, and anchoring force in VC. Figure 10 shows that the addition of BF effectively improves the peak shear strength. In the case of the same normal stress, the peak shear strength of samples D32, D22, and D13 increased by 79.21%, 52.73% and 38.9% respectively. This is because the introduction of BF increases the cohesion, friction, and anchoring force between the interfaces. Short length or a small amount of fibers don’t achieve the reinforcing effect. Too long too many fibers produce weak structure planes, which lead to the decrease of strength.

For the blank samples (i.e. D00), the peak and residual shear strength differences is between 74.6 kPa and 101.86 kPa. For the BF reinforced VC samples, the peak, and residual shear strength differences is between 48.8 kPa and 216.4 kPa (except D22 and D23). For the samples without BF content, the curves present a softening behavior. The softening degree of the blank samples is less than the fiber-reinforced samples. It is found that the BF mainly improves cohesion and anchoring force in VC. The residual shear strength of the basalt fiber-reinforced samples is higher than the blank samples, which indicates that the friction between particles of the samples is also improved by the addition of BF. The peak and residual shear strength of D22 (length = 9 mm, *w* = 0.35%) and D23 (length = 9 mm, *w* = 0.65%) are similar. This shows that even if the spraying strength of fiber-reinforced VC is reduced in practical engineering, it has little effect on its strength and durability.

#### Cohesion and internal friction Angle

**Figure 12:**
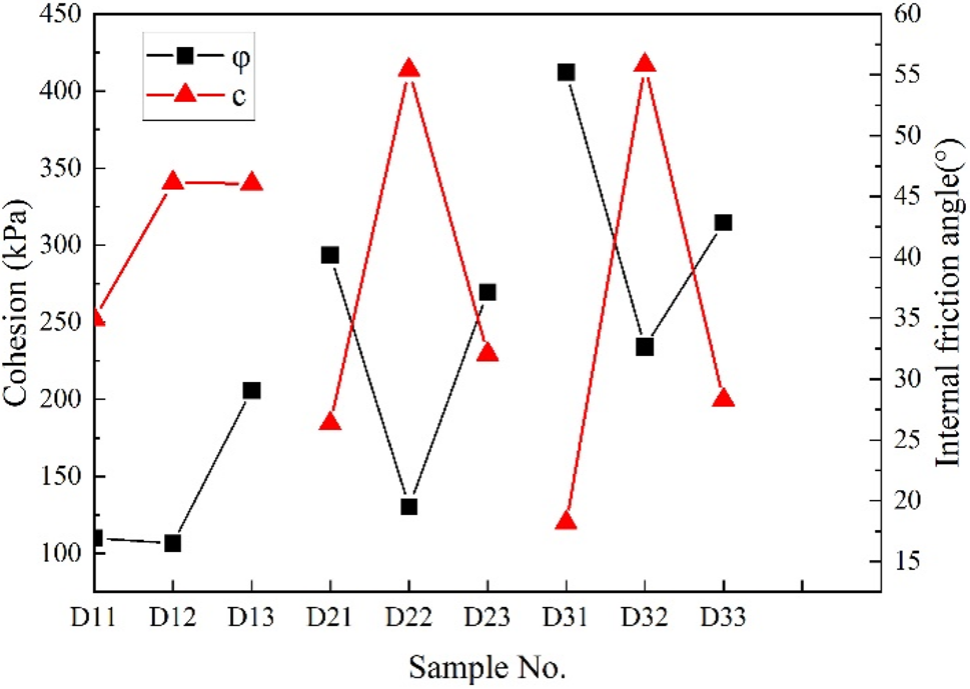
Curves on the cohesion and internal friction angle with different BF length and content.

Shear strength is important to evaluate the stability and durability of VC shows the relationship between the cohesion and internal friction angle of VC with different fiber lengths and content. The cohesion and internal friction angle of VC increase 70% and 58.55% respectively after adding BF. This shows that BF effectively improves the shear performance of VC. Figure 12 shows that the cohesion and internal friction angle of short fiber (length = 6 mm) increase with the increased content. Because the fiber is too short, the cohesion and anchoring number can only be increased by increasing the fiber number, resulting in increased cohesion. The cohesion and anchoring increase the particle size increases the energy required to move the shear process to a new position, and correspondingly increases the sliding resistance, thus increasing the internal friction angle.

However, the larger fiber (length = 9 mm and 12 mm) content is 0.35%, the cohesion is the largest, and the internal friction angle is the smallest. The test results show that is a negative correlation between the cohesion and the internal friction angle of VC, which is similar to that non-reinforced. The typical negative correlation of fiber-reinforced vegetation concrete is mainly the fiber anchors more cement hydration products to increase the anchoring force (expressed in cohesion), resulting in the reduction of large-size aggregates and the reduction of the internal friction angle.

The maximum cohesion of the sample under the three mixing ratios is when the fiber length is 9 mm. This verifies the accuracy of the formula for critical reinforcement length. Fiber length growth not only improves interfacial friction but also forms a network structure in the aggregate, which improves shear resistance. However, too long and too excess fibers instead form weak structural surfaces and reduce shear strength. The optimal reinforcement length of basalt fiber is 9 mm and the optimum content is 0.35% for VC.

## Conclusion

In this study, Single basalt fiber (BF) pullout tests were introduced into vegetation concrete (VC), and qualitatively analyzed on the interfacial shear strength of BF reinforced. Moreover, the accuracy of the optimal BF reinforcement length formula was verified by the direct shear tests. The following conclusions are obtained:

1. The pullout load–displacement curves show that the applied load increases quickly with increasing pullout displacement, which drops in a typical multi-peaks pattern after a peak load is reached. The anchoring action between cement hydration products and fibers in VC changes the mechanical action of BF/VC and results in this phenomenon.
2. Water content plays a key factor in BF/VC interfacial shear strength. Water content affects the interfacial shear strength by cement hydration, matrix suction, and effective stress between particles. Lower inter-particle density is greatly affected by cement hydration.
3. The interfacial shear strength of BF reinforced VC is significantly influenced by dry density. Higher dry density helps enhance interfacial mechanical interactions. The $\tau_{f}$ increases by 149.23% on average with the increase of dry density.
4. BF can significantly improve the peak and residual shear strength of vegetation concrete with proper BF length and content. At the optimal BF length and content, BF reinforced VC shows no obvious softening behavior.
5. 5The formula of optimal BF reinforcement length effectively calculates the optimum length of added fibers in VC. It is of practical engineering significance to provide economical fiber length.

## Data Availability

Some or all data, models, or code that support the findings of this study are available from the corresponding author upon reasonable request.

## References

[1] Cao S (2011). Impact of China's large-scale ecological restoration program on the environment and society in arid and semiarid areas of china: achievements, problems, synthesis, and applications. Crit. Rev. Environ. Sci. Technol..

[2] Cao S, Zhang J, Chen L, Zhao T (2016). Ecosystem water imbalances created during ecological restoration by afforestation in China, and lessons for other developing countries. J. Environ. Manage..

[3] Li B (2018). The primate extinction crisis in China: immediate challenges and a way forward. Biodivers. Conserv..

[4] Bi Y, Guo C, Wang K (2020). Research progress of biological improvement of reclaimed soil in coal mining area. Coal Sci. Technol..

[5] Hu Z (2019). The 30 yearsland reclamation and ecological restoration in China:review, rethinking and prospect. Coal Sci. Technol..

[6] Xu J, Zhu X, Yuan K (2019). Effects of different restoration measures on species diversity and aboveground biomass of the gold mining area in headwaters of the Ertix River. Arid Land Geography.

[7] Yin R, Yin G (2010). China's primary programs of terrestrial ecosystem restoration: initiation, implementation, and challenges. Environ. Manage..

[8] Chen F, Xu Y, Wang C, Mao J (2013). Effects of concrete content on seed germination and seedling establishment in vegetation concrete matrix in slope restoration. Ecol. Eng..

[9] Xu, Y. & Chen, F. in *Sustainable Cities Development and Environment, Pts 1–3* Vol. 209–211 *Applied Mechanics and Materials* (ed W. J. Yang) 1027–1031 (2012).

[10] Prabakar J, Sridhar RS (2002). Effect of random inclusion of sisal fibre on strength behaviour of soil. Constr. Build. Mater..

[11] Hejazi SM, Sheikhzadeh M, Abtahi SM, Zadhoush A (2012). A simple review of soil reinforcement by using natural and synthetic fibers. Constr. Build. Mater..

[12] Li VC (2002). Large volume, high-performance applications of fibers in civil engineering. J. Appl. Polym. Sci..

[13] Tang C, Shi B, Gao W, Chen F, Cai Y (2007). Strength and mechanical behavior of short polypropylene fiber reinforced and cement stabilized clayey soil. Geotext. Geomembr..

[14] Lee, C. H., Khalina, A., Lee, S. H., Liu, M. A. Comprehensive review on bast fibre retting process for optimal performance in fibre-reinforced polymer composites. *Adv. Mater. Sci. Eng.* **2020**, 10.1155/2020/6074063 (2020).

[15] Ferdous W, Bai Y, Almutairi AD, Satasivam S, Jeske J (2018). Modular assembly of water-retaining walls using GFRP hollow profiles: components and connection performance. Compos. Struct..

[16] Jiang S-F, Ma S-L, Wu Z-Q (2014). Experimental study and theoretical analysis on slender concrete-filled CFRP-PVC tubular columns. Constr. Build. Mater..

[17] Kim, K.-H. E., Andrawes, B. & Duarte, C. A. *Behavior of FRP retrofitted bridge timber piles under earthquake and tsunami loading*. (2017).

[18] Lin T-C, Jeng C-H, Wang C-Y, Jou T-H (2012). Repair of corroded prestressed concrete piles of harbor landing stages. ACI Struct. J..

[19] Reddy BP, Alagusundaramoorthy P, Sundaravadivelu R (2009). Retrofitting of RC piles using GFRP composites. KSCE J. Civ. Eng..

[20] Ghazavi M, Roustaie M (2010). The influence of freeze-thaw cycles on the unconfined compressive strength of fiber-reinforced. Cold Reg. Sci. Technol..

[21] Gullu H, Khudir A (2014). Effect of freeze-thaw cycles on unconfined compressive strength of fine-grained soil treated with jute fiber, steel fiber and lime. Cold Reg. Sci. Technol..

[22] Ghazavi M, Roustaei M (2013). Freeze-thaw performance of clayey soil reinforced with geotextile layer. Cold Reg. Sci. Technol..

[23] Gullu H, Hazirbaba K (2010). Unconfined compressive strength and post-freeze-thaw behavior of fine-grained soils treated with geofiber and synthetic fluid. Cold Reg. Sci. Technol..

[24] Ding M, Zhang F, Ling X, Lin B (2018). Effects of freeze-thaw cycles on mechanical properties of polypropylene Fiber and cement stabilized clay. Cold Reg. Sci. Technol..

[25] Orakoglu ME, Liu J (2017). Effect of freeze-thaw cycles on triaxial strength properties of fiber-reinforced clayey soil. KSCE J. Civ. Eng..

[26] Tang C-S, Shi B, Zhao L-Z (2010). Interfacial shear strength of fiber reinforced soil. Geotext. Geomembr..

[27] Khalil H, Ismail H, Rozman HD, Ahmad MN (2001). The effect of acetylation on interfacial shear strength between plant fibres and various matrices. Eur. Polymer J..

[28] Godara A (2010). Interfacial shear strength of a glass fiber/epoxy bonding in composites modified with carbon nanotubes. Compos. Sci. Technol..

[29] Hinoki T, Zhang W, Kohyama A, Sato S, Noda T (1998). Effect of fiber coating on interfacial shear strength of SiC/SiC by nano-indentation technique. J. Nucl. Mater..

[30] Medina MC (2016). Comparison of push-in and push-out tests for measuring interfacial shear strength in nano-reinforced composite materials. J. Compos. Mater..

[31] Gigante V (2017). Effects of waviness on fiber-length distribution and interfacial shear strength of natural fibers reinforced composites. Compos. Sci. Technol..

[32] Jamshaid H, Mishra R (2016). A green material from rock: basalt fiber - a review. J. Text. Inst..

[33] Katkhuda H, Shatarat N (2017). Improving the mechanical properties of recycled concrete aggregate using chopped basalt fibers and acid treatment. Constr. Build. Mater..

[34] Niu D (2019). Experimental study on mechanical properties and fractal dimension of pore structure of basalt-polypropylene fiber-reinforced concrete. Appl. Sci. Basel.

[35] Ramesh M, Palanikumar K, Reddy KH (2017). Plant fibre based bio-composites: sustainable and renewable green materials. Renew. Sustain. Energy Rev..

[36] Guo Y, Hu X, Lv J (2019). Experimental study on the resistance of basalt fibre-reinforced concrete to chloride penetration. Constr. Build. Mater..

[37] Sakthivel M, Jenarthanan M, Raja P (2019). Mechanical properties, degradation and flue gas analysis of basalt and glass fiber reinforced recycled polypropylene. Mater. Test..

[38] Wang X, Li C, Zhou W (2017). Experimental study on load-carrying capacity of basalt fiber reinforced concrete short columns under axial compression. Bull. Chin. Ceram. Soc..

[39] Kan, Z. B. & Li, Y. R. in *Architecture and urban development* Vol. 598 *Advanced Materials Research* (eds Z. H. Zhang & Y. J. Li) 627–630 (2012).

[40] Wang, H. L. & Zhong, Y. H. in *Research in materials and manufacturing technologies, Pts 1–3* Vol. 835–836 *Advanced Materials Research* (eds Y. H. Kim & P. Yarlagadda) 730–737 (2014).

[41] Wu X (2016). Experimental research on mechanical properties of basalt fiber reinforced lightweight aggregate concrete. Ind. Construct..

[42] Zhou H, Jia B, Huang H, Mou YL (2020). Experimental study on basic mechanical properties of basalt fiber reinforced concrete. Materials..

[43] Chao-sheng T, Bin SHI, Wei GAO, Jin LIU (2009). Single fiber pull-out test and the determination of critical fiber reinforcement length for fiber reinforced soil. Rock Soil Mech..

[44] Zhang C, Zhu H, Tang C, Shi B (2015). Modeling of progressive interface failure of fiber reinforced soil. J. Zhejiang Univ. Eng. Sci..

